# Miscellany

**Published:** 1905-02

**Authors:** 


### Miscellany.

New Appliances advertised in the English Dental Journals, 1904.—The Dental Manufacturing Company, of London, Manchester, and Dublin, offer the following:

A pure nickel flask, suggested by Mr. G. Brunton, for vulcanizing repairs, small dentures, and regulating appliances which can be made on the model, and which do not require the rubber to be packed under pressure. This is a method of constructing vulcanite pieces which has not been generally adopted on this side. With some repair cases flasking can be avoided by packing the rubber in place; small dentures and regulating appliances can be made by cementing the rubber onto the model, building it up into the desired form and shape, and after covering it with tin-foil embedding it in powdered talc or French chalk, and immediately placing it in the vulcanizer. This flask is designed for this purpose, and consists of a cup-shaped vessel made of pure nickel and provided with a tight-fitting cover held in place with spring clips. Mr. Brunton claims that this is much cleaner than flasking in plaster in the ordinary way, and that the quality of the finished product is better.

A renewal flask, suggested by Mr. Boucher. This is a gun-metal flask, square in shape, and very deep, and is especially designed for flasking cases nearly vertical to facilitate renewing the pink rubber rim representing the gum. Its shape and roominess make it a desirable addition to laboratory appliances.

The "accurate flask," suggested by Mr. W. Paton Scott. This flask, instead of the usual bolts, has side lugs on the upper half through which screws pass into the sides of the lower half of the flask. As these screws are in place when the flask is filled with plaster, and cannot be replaced after the case is packed unless the upper and lower sections are precisely in the same position as they were when the plaster was placed in, it is claimed that breaking of models by overpressure, or raising of bites from the flask not being completely closed is absolutely prevented. There is no means provided in the flask for closing it during packing; this is presumed to be done with some kind of a press.

The Brunton soldering appliance. This is a stand very much like a chemist's retort stand, supporting a large clay crucible fitted with a Bunsen burner, which is fed with gas through a rubber tube. The work to be soldered, inverted as is usual, is placed on a grid which rests inside the crucible over the flame. When ready for soldering the gas is lighted, the work placed in position, and a crucible cover placed over the crucible. When the case is sufficiently heated, the crucible, which rests in an iron ring attached to the stand, can be slightly raised and tilted to either side, thus enabling the blow-pipe to play on the parts to be soldered. When the soldering is completed the cover is replaced and the case allowed to cool down. It is a handy appliance, and easily made when it cannot be had, but the price at which it is offered is so close to that of the required material, it is economy to purchase.

The Parris lower molar crown, devised by Mr. Stanway Parris. This molar crown resembles a Logan crown without a dowel. The part fitting onto the root is provided with a large recessed cavity coinciding with the pulp-chamber of the tooth. It is held in position by a double-headed stud made by soldering two pieces of metal to a wire, or it may be turned from wire of sufficiently large diameter. The root is prepared by grinding off the remains of the crown level with the gum, and as flat as possible. The crown is then fitted so as to make a close joint. The pulp-chamber is enlarged to accommodate the stud, and recessed. The cavity in the root and that in the crown is filled with cement in which the stud is embedded, and the crown is then forced into place. The idea seems to be excellent for cases where it is inexpedient or inconvenient to adjust dowels to the pulp-canals. Whether the stud is a desirable addition may be questioned; so large a body of cement will in most cases be amply strong, while the stud, by becoming slightly displaced, or by failure to completely embed in the cement, may prove an element of weakness. The idea somewhat resembles one suggested by Dr. Horatio C. Merriam, Salem, Mass. (*Dental Cosmos*, vol. xxviii., August, 1886, page 493.)—W. H. TRUEMAN.

